# Purpura Hemorrhagica; with a Report of Cases

**Published:** 1859-01

**Authors:** G. W. Phillips

**Affiliations:** Dixon, Ill.


### PURPURA HEMORRHAGICA; WITH A REPORT OF CASES.

(Read at the Annual Meeting of the Illinois State Medical Society, June, 1858.)

BY G. W. PHILLIPS, M. D., OF DIXON, ILL.

The above is the title to an interesting paper covering thirty-five pages of foolscap paper; but the author must pardon us for giving an abstract instead of the paper in full, on account of the small amount of money in the treasury of the society. The whole paper is occupied with the detail of four cases and their treatment.

The first was a female, aged fifty years, who had suffered more or less from periodical fever for two years preceding. She was anemic, and the purpura was accompanied by hemorrhage from the bladder, and more or less bloody discharges from the bowels, with a plain aggravation of symptoms every alternate day. She was treated chiefly with quinine and oil of turpentine, and recovered in about three weeks.

The second case was a man, aged twenty years, of a scrofulous diathesis, and subject to attacks of pulmonary irritation. The symptoms, at first, were those of a mild remittent fever; but the patient became greatly prostrated; subject to frequent epistaxis; tongue dry; pulse 160 per minute; and towards the end of the case, petechia on the surface and low delirium. The treatment consisted in the use of quinine, brandy, ammonia and iron; but the patient died thirty-three days from the commencement of the attack. No post-mortem was allowed.

The third case was a female, of nervous temperament, aged between twenty and thirty years, whose health had been failing for two preceding years. During the last six or eight months she had nursed an infant, and suffered some from stomatitis materna, and slight attacks of periodical fever. The child was weaned, and the patient resorted for a time to a less malarious district in Wisconsin. While there her child died and she suffered from bronchitis. She returned emaciated and feeble, and soon again had symptoms of ague. For two weeks more she rapidly became extremely anemic, with vertigo, faintness on rising up; slight hemorrhages from the nose and gums; pulse 130 per minute and soft: and occasionally vomiting. There was enlargement of the spleen, and some signs of disease in the lungs. During the third week diarrhœa occurred, with some petechia over the abdomen. During the fourth week she began to improve, and remained better for several weeks. But then all the primary symptoms were renewed, with the addition of still greater emaciation, œdema of the cellular tissue, and dulness on percussion over the cardiac region of the stomach. After lingering eight or ten weeks longer, during which she suffered much from neuralgic pains and extreme debility, with additional dropsical effusions into the cavities of the body, she died.

Dr. Phillips thinks the case was complicated with cancerous disease of the stomach, but no post-mortem examination was permitted.

The fourth case was a lady, aged thirty-four years. Her father and some of her brothers had died of paralysis. The family on her mother's side were consumptive. Her health had been declining for more than a year before coming under Dr. Phillips' care. He found her suffering from all the symptoms usually resulting from occasional paroxysms of fever; greatly impoverished blood; enlargement of the spleen; and morbid irritability of the respiratory organs. For several months the patient continued to suffer from the same pathological conditions, with gradually increasing exhaustion. At length, epistaxis occurred, with petechial and purpuric spots over the cutaneous surface, varying from the size of a pin's head to that of a silver dollar. Owing to the frequently repeated symptoms of periodical fever, and the hereditary tendency to paralysis, she was treated during several weeks with strychnine and the solution of arsenite of potassa. These remedies were gradually increased until the former was given in doses of half a grain three times a day, and the latter in doses of fifteen drops, without spasmodic action from the one or œdema from the other. She took also more or less quinine and other tonics. After several months of suffering she improved so much as to be able to return to New Hampshire, her native state.

It will be observed that all the cases reported occurred in a miasmatic district, and that repeated attacks of periodical fever had preceded any hemorrhagic or purpuric symptoms.

The chief items of treatment that seemed to benefit the patients were such as interrupt periodicity and improve the quality of the blood, viz: the use of quinine, strychnine, nitro-muriatic acid, and other tonics.

